# Supplementary material for: MetaRibo-Seq measures translation in microbiomes
Source: Nat Commun. 2020 Jun 29;11:3268. doi: 10.1038/s41467-020-17081-z (PMC7324362; doi:10.1038/s41467-020-17081-z)
Supplement: Supplementary file 10 — Supplementary Data 7 [file 41467_2020_17081_MOESM10_ESM.zip › File2/Confidence_VeryHigh_Taxonomy/223071_out.krona.html]

Javascript must be enabled to view this page.

members
magnitude
magnitudeUnassigned
count
unassigned
taxon
rank

223071\_out

13

13
2
superkingdom

phylum
1239
13

class
13
186801

13
186802
order

family
31979
3

genus
3
1485

species
1262822
1

SRS014235\_contig\_number\_22547


SRS019685\_contig\_number\_32644SRS022524\_contig\_number\_7938
1262841
2
species

family
216572
1

genus
1
459786


SRS024435\_contig\_number\_27506
species
1
1897011

species
1898207
9

SRS020394\_contig\_number\_5278SRS020869\_contig\_number\_30627SRS024132\_contig\_number\_24816SRS064757\_contig\_number\_21326SRS098571\_contig\_number\_49074SRS1041133\_contig\_number\_contig-100\_256.257SRS146812\_contig\_number\_27892SRS147557\_contig\_number\_31002SRS150029\_contig\_number\_15065
